# Antibodies against human papillomavirus type 16 (HPV-16) and conjunctival squamous cell neoplasia in Uganda

**DOI:** 10.1038/sj.bjc.6600950

**Published:** 2003-06-10

**Authors:** K Waddell, J Magyezi, L Bousarghin, P Coursaget, S Lucas, R Downing, D Casabonne, R Newton

**Affiliations:** 1 Box 4008, Kampala, Uganda; 2 Laboratoire de Virologie Moléculaire, INSERM EMIU 00-10 and USC INRA, Faculté de Pharmacie, 37200 Tours, France; 3 Department of Histopathology, GKT School of Medicine, St. Thomas' Hospital, London, UK; 4 Centers for Disease Control and Prevention, Programme on AIDS, Uganda Virus Research Institute, PO Box 49, Entebbe, Uganda; 5 Cancer Research UK, Epidemiology Unit, Gibson Building, Radcliffe Infirmary, Oxford OX2 6HE, UK

**Sir**,

In a recent case–control study from Uganda, Newton *et al* (2002) reported the odds ratios for conjunctival carcinoma in relation to HPV-16 were 1.0 for anti-HPV-16 antibody negative (baseline group), 0.7 (0.2–2.9) for medium titre and 6.3 (1.2–33.4) for high-titre infection (*P*_trend_=0.2). It was concluded that there was insufficient evidence to support a role for HPV-16 in the aetiology of conjunctival cancer. We have now investigated the issue further in Uganda, using the same assay for HPV-16 antibodies as used in the earlier study. From November 1995 to May 2001, all patients with a provisional diagnosis of conjunctival squamous cell neoplasia who presented to a single surgeon (KW) in ophthalmology clinics throughout Uganda were recruited for study. After informed consent was obtained, tests for human immunodeficiency virus-1 (HIV) infection were offered and pretest counselling provided. Sociodemographic and clinical details were recorded from all the participants. HIV test results were reported back to the patients, together with post-test counselling and any remaining plasma was stored at minus 40°C. The study was approved by the Uganda National Council for Science and Technology and by the Science and Ethics Committee of the Uganda Virus Research Institute.

Appropriate treatment was provided to all the participants. Excised tumours were fixed in formal saline and sent to St Thomas' Hospital London for histopathological review by a single pathologist (SL). Conjunctival intraepithelial neoplasia (CIN) was classified into three stages as dysplasia occupying one-, two- or three-thirds of the epithelial thickness (CIN I–III; CIN III is synonymous with carcinoma *in situ*). Plasma samples were shipped on dry ice to the Laboratoire de Virologie Moléculaire, in Tours, France, where they were tested for antibodies against HPV-16, in a blinded fashion, using methods described elsewhere (Newton *et al*, 2002). Patient information and test results were recorded onto EPI-INFO (Dean *et al*, 1990) software and statistical analyses were conducted using STATA (STATA Corp., 2001).

From a total of 476 patients, 291 had enough stored plasma for anti-HPV-16 antibody testing, but following histological review, 37 of the 291 turned out to have diagnoses other than conjunctival neoplasia, such as pingueculae and inflammatory lesions. These individuals comprise the control group in analyses of the prevalence of anti-HPV-16 antibodies. The odds of anti-HPV-16 antibodies were compared between cases and controls, using odds ratios, estimated with unconditional logistic regression, adjusting for age group (<25, 25–34, 34+ years), sex and HIV serostatus.

The seroprevalence of HIV infection was 67% (169 of 254) among cases and 35% (13 of 37) among controls. The prevalence of antibodies against HPV-16 was 15% (37 of 254) among those with conjunctival neoplasia and 16% (six of 37) among controls (odds ratio 1.1, 95% confidence intervals 0.4–2.9). Table 1

**Table 1:** Prevalence of anti-HPV-16 antibodies among cases and controls

**Diagnosis**	**Percentage with anti-HPV-16 antibodies (number positive/total)**	**Percentage with medium titres of anti-HPV-16 antibodies (number positive/total)**	**Percentage with high titres of anti-HPV-16 antibodies (number positive/total)**
*All Subjects*			
Controls	16% (6/31)	14% (5/37)	3% (1/37)
Cases			
Total	15% (37/254)	8% (21/254)	6% (16/254)
CIN I	18% (5/28)	11% (3/28)	7% (2/28)
CIN II	15% (5/34)	12% (4/34)	3% (1/34)
CIN III	14% (12/84)	7% (6/84)	7% (6/84)
Invasive	14% (15/108)	7% (8/108)	6% (7/108)
			
*HIV-seronegative subjects*			
Controls	21% (5/24)	17% (4/24)	4% (1/24)
Cases			
Total	15% (13/85)	8% (7/85)	7% (6/85)
CIN I	8% (1/12)	0% (0/12)	8% (1/12)
CIN II	9% (1/11)	0% (0/11)	9% (1/11)
CIN III	17% (5/29)	10% (3/29)	7% (2/29)
Invasive	18% (6/33)	12% (4/33)	6% (2/33)
			
*HIV-seropositive subjects*			
Controls	8% (1/13)	8% (1/13)	0% (0/13)
Cases			
Total	14% (24/169)	8% (14/169)	
CIN I	25% (4/16)	19% (3/16)	6% (10/169)
CIN II	17% (4/23)	17% (4/23)	6% (1/16)
CIN III	13% (7/55)	5% (3/55)	0% (0/23)
Invasive	12% (9/75)	5% (4/75)	7% (4/55)

shows the prevalence of anti-HPV-16 antibodies according to the titre and the histological stage of conjunctival neoplasia, stratified by HIV serostatus. Table 2

**Table 2:** Summary of the association between a measure of anti-HPV-16 antibody titre and the risk of conjunctival neoplasia

	**HIV seronegative**	**HIV seropositive**	**All subjects**
**Anti-HPV-16 antibody status**	**Odds ratio and 95% confidence intervals^a^**	**Odds ratio and 95% confidence intervals^a^**	**Odds ratio and 95% confidence intervals^b^**
Seronegative	1.0	1.0	1.0
Seropositive–low titre	0.5 (0.1–1.9)	1.2 (0.1–10.7)	0.6 (0.2–2.0)
Seropositive–high titre	2.2 (0.2–20.8)	—	3.3 (0.4–27.6)
	*χ*^2^ trend (1 d.f.)=0.0	*χ*^2^ trend (1 d.f.)=0.8	*χ*^2^ trend (1 d.f.)=0.4
	*P*=0.9	*P*=0.4	*P*=0.5

a Odds ratios adjusted for age group (<25, 25–34, 34+years) and sex.

b Odds ratios adjusted for age group, sex and HIV serostatus. d.f.=degrees of freedom.

shows the odds ratio for conjunctival neoplasia associated with a measure of anti-HPV-16 antibody titre, stratified by HIV serostatus. We find no evidence of a statistically significant association between anti-HPV-16 antibody status and the risk of conjunctival neoplasia. Although its statistical power is low, this study supplements the information already reported by Newton *et al* (2002). Specifically designed larger studies offer most hope of identifying any underlying infectious cause of conjunctival neoplasia.
